# Effect of aging on the cerebral metabolic mechanism of electroacupuncture treatment in rats with traumatic brain injury

**DOI:** 10.3389/fnins.2023.1081515

**Published:** 2023-04-11

**Authors:** Bei-Bei Huo, Mou-Xiong Zheng, Xu-Yun Hua, Jia-Jia Wu, Xiang-Xin Xing, Jie Ma, Min Fang, Jian-Guang Xu

**Affiliations:** ^1^School of Rehabilitation Science, Shanghai University of Traditional Chinese Medicine, Shanghai, China; ^2^Engineering Research Center of Traditional Chinese Medicine Intelligent Rehabilitation, Ministry of Education, Shanghai, China; ^3^Department of Traumatology and Orthopedics, Yueyang Hospital, Shanghai University of Traditional Chinese Medicine, Shanghai, China; ^4^Department of Rehabilitation Medicine, Yueyang Hospital, Shanghai University of Traditional Chinese Medicine, Shanghai, China; ^5^Shuguang Hospital, Shanghai University of Traditional Chinese Medicine, Shanghai, China

**Keywords:** metabolic mechanism, traumatic brain injury (TBI), aging, small animal ^18^F-FDG PET/CT, electroacupuncture, brain plasticity

## Abstract

**Objective:**

Aging has great influence on the clinical treatment effect of cerebrovascular diseases, and evidence suggests that the effect may be associated with age-related brain plasticity. Electroacupuncture is an effective alternative treatment for traumatic brain injury (TBI). In the present study, we aimed to explore the effect of aging on the cerebral metabolic mechanism of electroacupuncture to provide new evidence for developing age-specific rehabilitation strategies.

**Methods:**

Both aged (18 months) and young (8 weeks) rats with TBI were analyzed. Thirty-two aged rats were randomly divided into four groups: aged model, aged electroacupuncture, aged sham electroacupuncture, and aged control group. Similarly, 32 young rats were also divided into four groups: young model, young electroacupuncture, young sham electroacupuncture, and young control group. Electroacupuncture was applied to “Bai hui” (GV20) and “Qu chi” (LI11) for 8 weeks. CatWalk gait analysis was then performed at 3 days pre- and post-TBI, and at 1, 2, 4, and 8 weeks after intervention to observe motor function recovery. Positron emission computed tomography (PET/CT) was performed at 3 days pre- and post-TBI, and at 2, 4, and 8 weeks after intervention to detect cerebral metabolism.

**Results:**

Gait analysis showed that electroacupuncture improved the forepaw mean intensity in aged rats after 8 weeks of intervention, but after 4 weeks of intervention in young rats. PET/CT revealed increased metabolism in the left (the injured ipsilateral hemisphere) sensorimotor brain areas of aged rats during the electroacupuncture intervention, and increased metabolism in the right (contralateral to injury hemisphere) sensorimotor brain areas of young rats.

**Results:**

This study demonstrated that aged rats required a longer electroacupuncture intervention duration to improve motor function than that of young rats. The influence of aging on the cerebral metabolism of electroacupuncture treatment was mainly focused on a particular hemisphere.

## Introduction

With economic growth and medical improvements, we are undergoing a profound demographic transition to an aging population. This change has been a serious social problem and has placed a huge economic burden on governments. During the aging process, the brain is often accompanied by a series of alterations in neurochemistry, structure, and function (Grady, 2012). Studies have found that aging is the most crucial risk factor for many cerebrovascular diseases such as traumatic brain injury (TBI) and stroke. Aged individuals tend to show unsatisfactory clinical outcomes and higher mortality rates than those of younger patients. Therefore, identifying a potential relationship between aging and disease prognosis is increasingly important.

This study focused on TBI, which is a major cause of death in trauma patients in China (Wu et al., 2008; Li et al., 2015). According to the US Centers for Disease Control and Prevention, approximately 2.5 million people suffered head injuries in 2010. The most common causes were falls (35%) and motor vehicle collisions (17%), with a total mortality rate of approximately 20–30% (Vella et al., 2017). Moreover, the incidence of TBI may continue to increase with increasing age and population density (GBD 2016 Traumatic Brain Injury and Spinal Cord Injury Collaborators, 2019). Despite incremental advances in neurosurgery, TBI still results in long-term disability and low quality of life (Jones et al., 2018; Liu J. et al., 2018). In clinical practice, particularly in elder patients, the rehabilitation effect is poor (Wang W. et al., 2015; Engeroff et al., 2018).

Evidence shows that brain plasticity is an important mechanism for functional recovery after central nerve injury, especially for motor function improvement, which is closely related to brain remodeling (Calautti et al., 2001; Feydy et al., 2002). However, brain plasticity significantly declines with increasing age, greatly reducing the therapeutic effect. Therefore, increasing attention has been paid to the importance of targeted and age-differential rehabilitation interventions and neurorehabilitation nursing (Corrigan et al., 2014). However, the available strategies applied to TBI are limited and non-individualized (Marklund et al., 2019).

In addition to conventional rehabilitation methods, acupuncture, an important component of Chinese traditional medicine, is used to treat age -related brain disorders such as stroke and Alzheimer’s disease (Chang et al., 2018; Feng et al., 2019; Li et al., 2019). Studies have found that the mechanism of acupuncture treatment is associated with brain plasticity (Chavez et al., 2017; Xiao et al., 2018), however, the age-related metabolic mechanism of acupuncture action remains unclear (Wang F. et al., 2015; Chavez et al., 2017). With the progress of neuroimaging techniques, non-invasive and high-resolution detection methods, such as positron emission computed tomography (PET/CT), can monitor the rate of glucose metabolism in the brain by ^18^F-fluorodeoxyglucose (^18^FDG), which is marked by the radionuclide ^18^F (Sperry et al., 2018). It is frequently used to investigate the degree of neural activity in the brain and to explore cerebral metabolism mechanisms.

In summary, it is essential to study the compensation mechanism of the brain in response to brain injury from the perspective of aging. In the current study, small-animal PET/CT was applied to both aged and young rats with TBI treated using electroacupuncture. We aimed to explore the effect of aging on the recovery of motor ability and the longitudinal cerebral metabolic mechanism of electroacupuncture treatment in rats with TBI and to provide new evidence for developing age-specific rehabilitation strategies in the clinic.

## Materials and methods

### Animals

Study rats were obtained from the Laboratory Animal Limited Liability Company of Slack (Shanghai, China). Rats were kept in a constant environment with a suitable temperature (21–23^°^C) and a 12 h light/dark cycle. Sufficient food and water were provided. To allow the rats to adapt to the environment, no manipulation was performed 1 week before any intervention.

Thirty-two healthy clean-grade aged female SD rats (18 months old) were randomly divided into four groups (*n* = 8/group): aged model (MA), aged electroacupuncture (EA), aged sham electroacupuncture (sham EA), and aged control (CA) group. Thirty-two healthy clean-grade young female SD rats (8 weeks old) were randomly divided into four groups (*n* = 8/group): young model (MY), young electroacupuncture (EY), young sham electroacupuncture (sham EY), and young control (CY) group.

All procedures were conducted according to the Guide for the Care and Use of Laboratory Animals described by the U. S. National Institutes of Health. The protocol was approved by the Animal Ethical Committee of Shanghai University of Traditional Chinese Medicine (No. PZSHUTCOM190712007).

### TBI model

**FIGURE 1:**
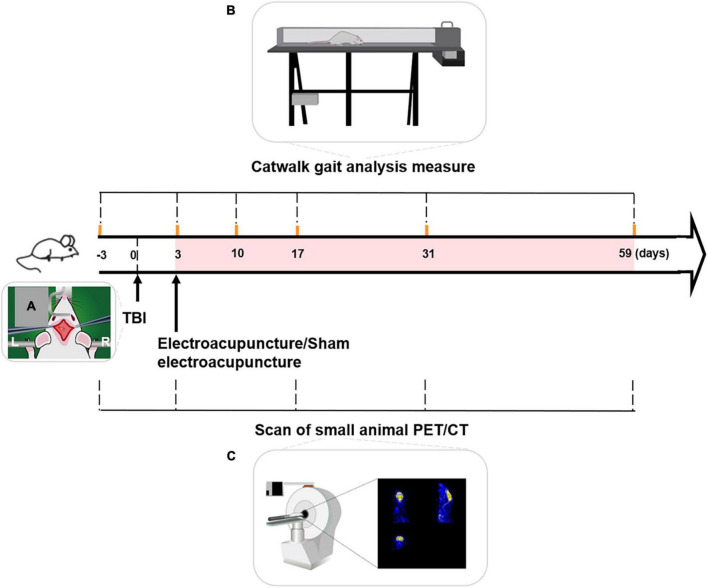
Schematic process of experiment. **(A)** TBI model with left motor cortex injury was established by controlled cortical impact (CCI). **(B)** Timeline of Catwalk gait analysis measure over the experiment. Catwalk gait tests of the right forepaw (forepaw affected by left-sided TBI) were conducted at baseline and 3, 10, 17, 31, and 59 days after TBI. **(C)** Timeline of small animal PET/CT (positron emission tomography-computed tomography) scanning over the experiment. PET/CT scanning of rats’ brain was performed at baseline and on days 3, 17, 31, and 59, following TBI. Duration of electroacupuncture or sham electroacupuncture was marked with the pink shadow. TBI, traumatic brain injury, L, left, R, right.

Controlled cortical impact (CCI) was used to establish a TBI rat model. All rats were injected intraperitoneally with sodium pentobarbital at a dose of 40 mg/kg. After hair removal and iodine-volt disinfection, rat was fixed on a heated operating table. A longitudinal incision was made along the cranial midline to expose the left side of the skull. The location of the craniotomy area in the left cortex was 5 mm lateral to the midline, 4.5 mm anterior to bregma to 0.5 mm posterior to bregma. Coordinates were ML = 2.5 mm and AP = 2 mm. A hand-grinding drill was used to open a 5 mm diameter skull window to expose the dura mater (Hua et al., 2016). A 5 mm diameter impact rod was selected. The impact parameter settings were: impact depth = 2.8 mm, impact speed = 4 m/s, and residence time = 150 ms. After impact, the skin was sutured, and the TBI model was established (Figure 1A).

### Interventions

Electroacupuncture or sham electroacupuncture was performed 3 days after TBI in a quiet environment. The rat was immobilized on a custom-made fixator to expose the head and upper limbs.

The electroacupuncture intervention was as follows: acupuncture needles were inserted into the acupoint of “Bai hui” (Mao et al., 2020) and right “Qu chi” (Chen et al., 2012), respectively. An electroacupuncture apparatus was used to output a dense dispersion wave of 1/20 Hz. The stimulus intensity was adjusted until slight muscle convulsions occurred. The electroacupuncture intervention was set at 20 mins/day, 5 days/week, for 8 weeks. The rats underwent adaptation training with electroacupuncture before the first treatment.

Sham electroacupuncture involved different electroacupuncture acupoints than that of electroacupuncture intervention. Acupoints for sham electroacupuncture were 5 mm lateral to the acupoints of “Bai hui” (GV20) and right “Qu chi” (LI11). The electroacupuncture apparatus did not produce an electrical output.

The control and model groups did not receive any treatment.

### CatWalk gait analysis measurement

CatWalk gait analysis (CatWalk XT gait analysis system; Noldus, Wageningen, the Netherlands) of the right forepaw was performed at baseline and 3, 10, 17, 31, and 59 days after TBI (Figure 1B).

The CatWalk system consists of mainframe computers and software, and tests the core part of the runway. The runway is composed of a 1.3 m long glass platform and two movable baffles on both sides. At testing onset, the rat ran from one end of the track to the other. Once the forepaw touched the glass platform, the motion parameters of the rat pawprint were quickly captured using a high-speed camera (Figure 1B). The computer automatically identified and marked the prints for subsequent data analysis. All rats were trained before measurement of CatWalk gait analysis.

### PET/CT scanning procedure

PET/CT was performed at baseline and on days 3, 17, 31, and 59, following TBI (Figure 1C). For scan preparation, at each time point, food was withheld overnight to ensure that the radioactive tracer (^18^F-FDG) was fully enriched in the rat brain during scanning.

Positron emission computed tomography scanning procedure was as follows. The body weight of the rats was recorded to calculate the dose of ^18^F-FDG administered intravenously. A 40 min wait was necessary after the injection to ensure full absorption. Imaging acquisition was performed using a small-animal PET/CT scanner (Siemens Inc., Erlangen, Germany). An animal anesthetizing evaporator was used to anesthetize rats with halothane gas (dose of 5%). The rat was then placed in a prone position on the scanning bed. A dose of 1.5% halothane gas was used continuously. After attenuation correction, PET images (axial, coronal, and sagittal views) of the rats were reconstructed using the OSEM3D mode. The images were acquired using the following parameters: current, 500 μA; spherical tube voltage, 80 kV; and CT time, 492 s.

### Processing of PET images

The pre-processing and analytical procedures for PET/CT data were based on the MATLAB 2014a platform (Mathworks, Inc., Natick, MA, USA) and the Statistical Parametric Mapping 8 (SPM8) toolbox.^1^ First, the raw images were converted to the NIFIT format using ImageJ software (National Institutes of Health, Bethesda, MD, USA). Second, the voxels of the images were scaled with a 10x factor to ensure that the adequate algorithm was implemented in SPM8 (Tambalo et al., 2015). PET/CT images were reformatted to isometric voxels (2 × 2 × 2 mm^3^) and normalized to a standardized rat brain template. Finally, spatial smoothing was performed using a Gaussian kernel of 4 mm full width at half maximum.

### Statistical analysis

SPSS software (version 22.0; SPSS Inc.) was used to analyze CatWalk gait. The data are presented as mean ± standard deviation (SD). The mean intensity (MI) data were selected as indicators and analyzed with repeated measures analysis of variance (ANOVA) at different time points. The least significant difference (LSD) *post-hoc* test was used to compare the two groups. Statistical significance was set at *p* < 0.05.

Standard uptake values (SUVs) of ^18^F-FDG were selected as metabolic indicator. After preprocessing the PET/CT data, statistical analysis of the PET images was performed using the SPM 8, and a two-sample *t*-test was used to calculate changes in SUVs of ^18^F-FDG among different groups on days 17, 31, and 59 following TBI. The significance level was set at *p* < 0.01 and k > 20 voxels (En-Tao et al., 2013).

## Results

### CatWalk gait analysis measurement

MI of the right forepaw (forepaw affected by left-sided TBI) was selected for observation of functional recovery.

#### MI of the right forepaw in aged rat groups

**FIGURE 2:**
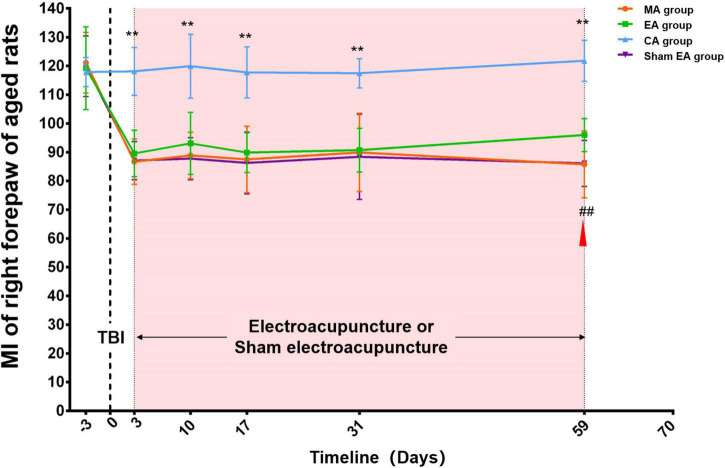
Catwalk gait analysis of the right (injured) forepaw in aged rat groups. At baseline, there was no difference among the four groups (MA, EA, CA, and sham EA groups) (*p* > 0.05). After TBI, the MI of right forepaw of aged rats in MA, EA and sham EA groups showed an obvious decrease compared with that in the CA group. During the subsequent observation process, there was an increase in these three groups. Throughout the experimental procedure, the MI of the right forepaw in the CA group was stable and significantly higher than that in the MA group (*p* < 0.01). After 8 weeks (day 59 post-TBI) of intervention, the MI of aged rat right forepaw in the EA group increased significantly compared with that in the sham EA group (*p* < 0.01). TBI establishment was showed by the dotted line. Mean and SD were expressed by the points and bars. The duration of electroacupuncture or sham electroacupuncture intervention was marked out with the pink shadow. 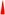 The red triangle represented the starting point of electroacupuncture for improving the aged rats’ MI. MA group, aged model group; EA group, aged electroacupuncture group; CA group, aged control group; sham EA group, aged sham electroacupuncture group; TBI, traumatic brain injury; MI, the mean intensity. **Represented a significant difference between the CA and MA groups at the same time point (*p* < 0.01). ##Represented a significant difference between the EA and sham EA groups at the same time point (*p* < 0.01).

At baseline, there was no difference among the four groups (MA, EA, sham EA, and CA group) (*p* > 0.05). After TBI, the MI of the right forepaw of aged rats in the MA, EA, and sham EA groups showed an obvious decrease compared with that in the CA group. During the subsequent observation process, there was an increase in these three groups. Throughout the experimental procedure, the MI of the right forepaw in the CA group was stable and significantly higher than that in the MA group (*p* < 0.01). The rats in the EA/sham EA group received electroacupuncture/sham electroacupuncture intervention on day 3 after TBI. After 8 weeks (day 59 post-TBI) of intervention, the MI of the aged rat right forepaw in the EA group increased significantly compared with that in the sham EA group (*p* < 0.01) (Figure 2).

#### MI of the right forepaw in young rat groups

**FIGURE 3:**
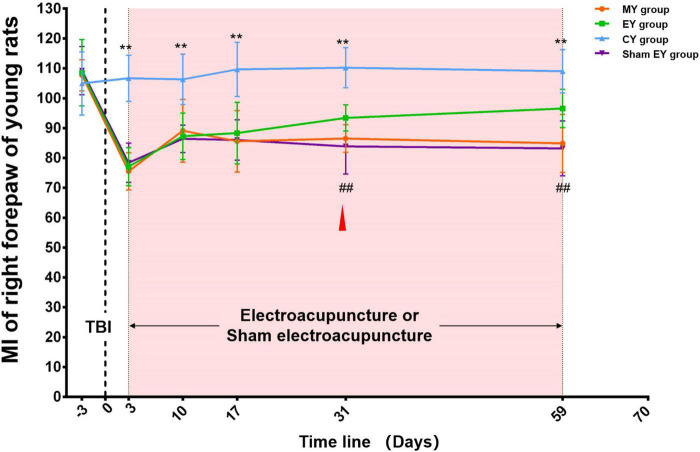
Catwalk gait analysis of the right (injured) forepaw in young rat groups. At baseline, there was no difference among the four groups (MY, EY, sham EY, and CY groups) (*p* > 0.05). After TBI, the MI of right forepaw of young rats in MY, EY, and sham EY groups showed an obvious decrease compared with that in the CY group. During the subsequent observation process, there was an increase in these three groups. Throughout the experimental procedure, the MI of the right forepaw in the CY group was stable and significantly higher than that in the MY group (*p* < 0.01). After 4 weeks (day 31 post-TBI) of intervention, the MI of right forepaw of young rats in the EY group increased significantly compared with that in the sham EY group (*p* < 0.01). TBI establishment was showed by the dotted line. Mean and SD were expressed by the points and bars. The duration of electroacupuncture or sham electroacupuncture intervention was marked out with the pink shadow. 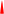 The red triangle represented the starting point of electroacupuncture for improving the young rats’ MI. MY group, young model group; EY group, young electroacupuncture group; CY group, young control group; sham EY group, young sham electroacupuncture group. TBI, traumatic brain injury; MI, the mean intensity. **Represented a significant difference between the CY and MY groups at the same time point (*p* < 0.01). ##Represented a significant difference between the EY and sham EY groups at the same time point (*p* < 0.01).

At baseline, there was no difference among the four groups (MY, EY, sham EY, and CY group) (*p* > 0.05). After TBI, the MI of the right forepaw of young rats in the MY, EY, and sham EY groups showed an obvious decrease compared with that in the CY group. During the subsequent observation process, there was an increase in these three groups. Throughout the experimental procedure, the MI of the right forepaw in the CY group was stable and significantly higher than that in the MY group (*p* < 0.01). The rats in the EY/sham EY group received electroacupuncture/sham electroacupuncture intervention on day 3 after TBI. After 4 weeks (day 31 post-TBI) of intervention, the MI of the right forepaw of young rats in the EY group increased significantly compared with that in the sham EY group (*p* < 0.01) (Figure 3).

### Brain metabolism

#### Changes of cerebral glucose metabolism after TBI in aged rat groups

**FIGURE 4:**
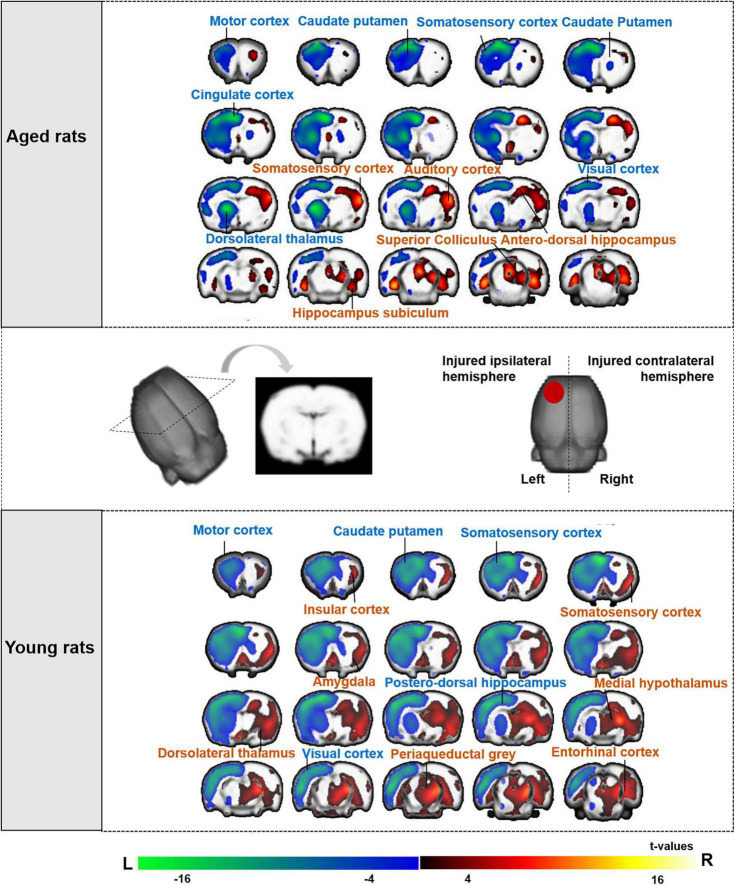
Brain regions of aged and young rat showing significant differences in metabolic activities after TBI compared with baseline. The changes of aged rats’ cerebral glucose metabolism after TBI were presented in the top of figure, and the changes of young rats were presented in the bottom of figure. Both aged and young rats showed extensively decreased metabolism on left (the injured ipsilateral) hemisphere after TBI. The warm color on brain region showed significant higher metabolic activities after TBI compared with baseline, while cold color showed significant lower metabolic activities after TBI compared with baseline. (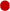) Red circle on rat brain showed the injured (left) hemisphere. TBI, traumatic brain injury; L, left, R, right.

We compared the SUVs of aged rats between pre- and post-TBI. The left (injured ipsilateral) hemisphere showed extensively decreased metabolism at 3 days post-TBI compared to baseline in the caudate putamen, motor cortex, somatosensory cortex, cingulate cortex, dorsolateral thalamus, and visual cortex. The right (contralateral to the injury) hemisphere showed increased metabolism at 3 days post-TBI compared to that at baseline in the somatosensory cortex, mesencephalic region, hippocampus subiculum, antero-dorsal hippocampus, superior colliculus and auditory cortex (The top of Figure 4).

#### Changes of cerebral glucose metabolism after TBI in young rat groups

We also compared the SUVs of young rats between pre- and post-TBI. Similarly, the left (injured ipsilateral) hemisphere showed extensively decreased metabolism at 3 days post-TBI compared to baseline in the motor cortex, caudate putamen, somatosensory cortex, postero-dorsal hippocampus and visual cortex. The right (contralateral to the injury) hemisphere showed increased metabolism at 3 days post-TBI compared to that at baseline in the somatosensory cortex, periaqueductal gray, insular cortex, dorsolateral thalamus, amygdala, entorhinal cortex, medial hypothalamus and medial geniculate (The bottom of Figure 4).

#### Changes in cerebral glucose metabolism after 2, 4, and 8 weeks electroacupuncture treatment in aged rats

For electroacupuncture intervention for 2 weeks, we compared the SUVs of rats between the EA and sham EA groups. Both the left (injured ipsilateral) and right (contralateral to injury) hemispheres showed decreased metabolism in the left somatosensory cortex, caudate putamen, and corpus callosum.

**FIGURE 5:**
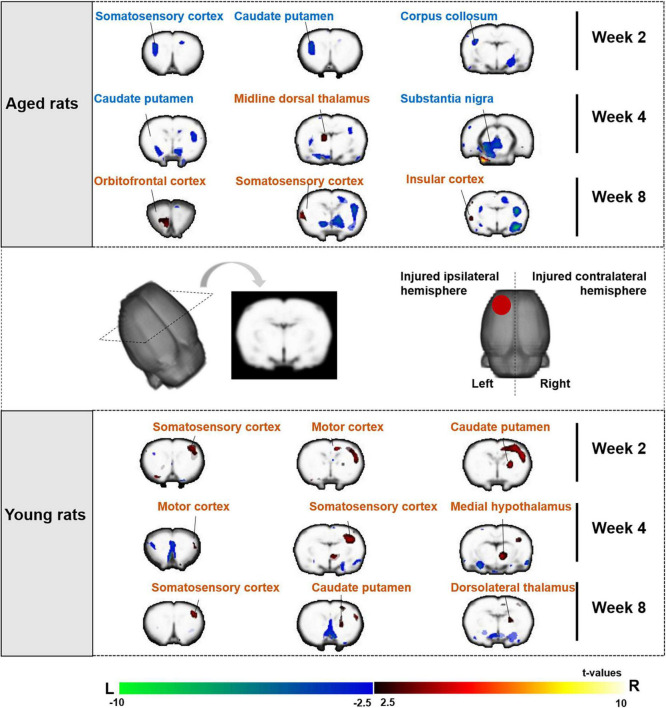
Sensorimotor brain regions of aged and young rats showing significant differences in metabolic activities on week 2, 4, and 8 after electroacupuncture intervention. The changes of aged rats’ cerebral glucose metabolism after 2, 4, and 8 weeks electroacupuncture were presented in the top of figure, and the changes of young rats were presented in the bottom of figure. The increased metabolism was in the left (the injured ipsilateral hemisphere) sensorimotor brain areas of aged rats during the electroacupuncture intervention, and the increased metabolism was in the right (contralateral to injury hemisphere) sensorimotor brain areas of young rats. The warm color on brain region showed significant higher metabolic activities in the EA/EY group compared with that in the sham EA/EY group, while cold color showed significant lower metabolic activities in the EA/EY group compared with that in the sham EA/EY group. EA group, the aged electroacupuncture group; sham EA group, the aged sham electroacupuncture group; EY group, the young electroacupuncture group; sham EY group, the young sham electroacupuncture group, (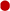) red circle on rat brain showed the injured (left) hemisphere, L, left, R, right.

Electroacupuncture intervention lasted for 4 and 8 weeks. Electroacupuncture mainly acted on the left (the injured ipsilateral) hemisphere in the aged rats, showing increased metabolism in the left sensorimotor brain area, including the somatosensory cortex, midline dorsal thalamus, orbitofrontal cortex and insular cortex, and decreased metabolism in the caudate putamen and substantia nigra (The top of Figure 5 and Table 1).

**TABLE 1:** Sensorimotor brain regions of aged and young rat significant metabolism changes in metabolic activities on week 2, 4, and 8 after electroacupuncture intervention.

Timeline	Metabolism changes EA-group vs. sham-EA group	Brain regions	MNI coordinates			Extent	*t*-value
			**x**	**y**	**z**		
**Aged rats**							
Week 2	Negative	Caudate putamen	–46	–3	–37	710	–4.789
		Corpus callosum	–40	9	–57	282	–4.669
		Somatosensory cortex	–63	22	–17	26	–4.207
Week 4	Positive	Midline dorsal thalamus	–15	–3	–19	148	3.219
	Negative	Substantia nigra	–19	–34	9	277	–5.464
		Caudate putamen	–30	–34	–49	225	–3.409
Week 8	Positive	Orbitofrontal cortex	–24	1	21	243	2.721
		Somatosensory cortex	–67	–7	–43	165	4.057
		Insular cortex	–65	–22	–27	104	3.264
**Young rats**							
Week 2	Positive	Somatosensory cortex	57	–12	–21	2,628	6.302
		Caudate putamen	55	–12	–19	976	6.794
		Motor cortex	9	44	–23	189	4.513
Week 4	Positive	Medial hypothalamus	14	–22	–5	561	4.604
		Somatosensory cortex	67	–1	–49	502	5.116
		Motor cortex	16	40	–49	26	3.743
Week 8	Positive	Somatosensory cortex	59	11	–21	684	5.667
		Caudate putamen	26	–3	–45	72	4.122
		Dorsolateral thalamus	26	–5	–31	26	3.156

Positive indicated EA group > sham EA group, and negative indicated EA group < sham EA group. *p* < 0.01.

#### Changes in cerebral glucose metabolism after 2, 4, and 8 weeks electroacupuncture treatment in young rats

For electroacupuncture intervention for 2 weeks, we compared the SUVs of rats between the EY and sham EY groups. Electroacupuncture mainly acted on the right (contralateral to injury) hemisphere in the young rats, showing increased metabolism in the right hemisphere including motor cortex, caudate putamen and somatosensory cortex.

Electroacupuncture intervention lasted for 4 and 8 weeks. Electroacupuncture mainly acted on the right (contralateral to injury) hemisphere in the young rats, showing increased metabolism in the right sensorimotor brain area, including caudate putamen, somatosensory cortex, motor cortex, medial hypothalamus and dorsolateral thalamus (The bottom of Figure 5 and Table 1).

## Discussion

This study investigated the effects of aging on the electroacupuncture intervention, including the recovery of motor function and underlying cerebral metabolic mechanisms. The TBI rat model was used as a representative of susceptible cerebrovascular disease in the elderly. Previous research indicated that TBI is a complex pathology often accompanied by impairment in physical functions, daily life ability, and/or cognitive abilities (Bland et al., 2011; O’Neil-Pirozzi et al., 2018). In the present study, the CCI model, which is one of the most common animal models of TBI, was selected. This model is relatively flexible in terms of the location and extent of brain injury. Other animal studies have found that electroacupuncture significantly improves nerve impairment in a TBI model (Gu et al., 2020). However, the impact of aging relative to the electroacupuncture interventions on brain plasticity is unknown. Therefore, in the current study, electroacupuncture was applied for 8 weeks to enable observation of motor recovery and further investigation of the effect of electroacupuncture interventions on brain remodeling of both aged and young TBI rats.

In behavioral assessment, the CatWalk rodent gait analysis system is a complete system for assessing movement defects or changes in gait caused by pain in animals (Koopmans et al., 2005). Presently, it is widely used in the research of neurological diseases such as brain injury, neuropathic pain, spinal cord injury, and peripheral nerve injury (Neumann et al., 2009; Pitzer et al., 2016; Liu Z. et al., 2018). MI is often used as an analysis parameter in rat brain injury models (Cross et al., 2015; Liu Z. et al., 2018). Our results showed that electroacupuncture could significantly increase the MI and improve motor function of the affected forepaw of TBI model rats in both the aged and young rat groups. However, aged rats required a longer electroacupuncture intervention than that in young rats. Brehmer et al. (2007) performed a memory-related study in humans and found that under the same memory training, elderly people showed considerable baseline plasticity which increased in the early stage of memory. However, their continued plasticity was reduced relative to that of young people. The brain experiences structural and functional deterioration with age, which might be associated with age-related decline in sensory motor plasticity (Faulkner et al., 2007; Goble et al., 2009). Although normal aging is associated with decreased brain function, brain plasticity is at least partially preserved in older adults (Chollet, 2013). Intuitively, we can supposed that aging reduces the brain’s capacity to reorganize against lesion (Chollet, 2013). Those may be related to the aged requiring longer intervention stimulation.

Glucose metabolism is the main source of energy in the brain and is closely related to neural activity (Passow et al., 2015). Therefore, PET is widely used to observe the cerebral distribution of glucose metabolism and to explore the mechanism of acupuncture treatment in the brain (Fang et al., 2012). We found that glucose metabolism was significantly decreased in the injured ipsilateral (left) hemisphere including the motor cortex, caudate putamen, somatosensory cortex, and significantly increased in the contralateral (right) hemisphere after brain injury, indicating that local injury had a wide impact on brain metabolism and caused redistribution of metabolic activities between the bilateral hemispheres. The complex balance between the hemispheres was broken by the brain injury. Consistent with previous findings, (García-Panach et al., 2011) found that differences in brain glucose metabolism in severe TBI were associated with neurological function, showing that the more severe the condition, the lower the metabolism. Moreover, reduced metabolism was associated with poorer outcomes. Zhang et al. (2010) studied the brain glucose metabolic activities of 81 patients with TBI and 68 normal controls using PET. The results showed significantly lower FDG uptake in the patients with TBI than that in normal controls. Decreased FDG uptake was widely distributed in the cortex (including the bilateral frontal and temporal regions) and the thalamus. Liang et al. (2018) investigated brain metabolism in middle cerebral artery occlusion (MCAO) rats using a PET scan and showed that metabolic decline occurred mainly in the injured ipsilateral hemisphere. These results imply that functional deficits in rats with TBI are mainly related to the injured ipsilateral hemisphere.

Previous studies have found that electroacupuncture stimulation can activate the corresponding brain regions, demonstrating a central effect of electroacupuncture. Evidence has revealed that rehabilitation therapies improve motor function after chronic stroke, generally relying on promoting plasticity in the cortex and other brain structures (Rossini et al., 2003; Oujamaa et al., 2009). In hemiplegic patients, the changes in movement were associated with activation or inactivation of brain motor network areas (Chollet, 2013). The motor cortex is critical for functional recovery. Other studies emphasize the activation of the early motor cortex, with a good prognosis. Our results showed in the preliminary stage (after electroacupuncture intervention for 2 weeks), electroacupuncture increased metabolism in the right hemisphere sensorimotor areas of young rat including motor cortex, caudate putamen and somatosensory cortex. The results were not found in the aged rat. Studies have shown that the improvement in energy metabolism is considered to be the mechanism of functional recovery after cerebral ischemia (Shen et al., 2013).

The excitability of the affected and unaffected hemispheres has been in controversy. Usually, we pay more attention to the injury side. Schaechter et al. (2007) performed acupuncture on patients with chronic hemiplegia for 10 weeks, and the results showed that acupuncture improved the function and range of motion of the affected upper limb, and there was a significant positive correlation with the active brain area on the healthy (uninjured) side. Kim et al. (2004) conducted a study on patients with left hemiplegia using functional magnetic resonance imaging (fMRI) and found that the sensorimotor areas of the bilateral hemisphere were activated when the patient’s involved hand moved, indicating that the healthy hemisphere played an important role in the process of functional recovery. In addition, a PET study showed that electroacupuncture can activate the bilateral hemispheric motor-related brain regions in patients with stroke. It is believed that electroacupuncture contributed to brain motor plasticity after stroke (Fang et al., 2012). Growing evidence from fMRI studies indicates that the healthy (uninjured) cortex is essential for inter-hemispheric plasticity (Kim et al., 2003; Pelled et al., 2007). Our study found that the main activated brain areas were concentrated in the left (injured) hemisphere after electroacupuncture intervention in aged rats and in the right (contralateral to injury) hemisphere after electroacupuncture intervention in young rats. These results might be of great significance for evidence-based guidelines and the prevention of sensorimotor deficits typically associated with the aging process. Both bilateral hemispheres should be valued.

In addition, there were some limitations in this study. Frist, we selected 18 months-old rats to investigate the effect of aging on brain remodeling. Future studies should consider grouping rats older than 18 months into consecutive age stages (e. g., 18, 21, 24, 30 months, etc.) and covering multiple time points in the aging process, which will allow them to detect more age-dependent metabolic changes in the brain. Secondly, in the further aging study, the molecular and protein detection methods must be included in order to elucidate the important role of brain metabolism in the improvement of motor function after TBI.

## Conclusion

Overall, the present study demonstrated that electroacupuncture could can improve motor function of the affected limb in both aged and young rats; however, aged rats required a longer intervention duration than that of young rats. The influence of aging on the cerebral metabolism of electroacupuncture treatment was mainly focused on a particular hemisphere. The effect of electroacupuncture on aged rats was manifested in the increase in the cerebral metabolism of sensorimotor-related brain areas in the left (injured ipsilateral) hemisphere, while the effect on young rats was manifested in the increase in the cerebral metabolism of sensorimotor-related brain areas in the right (contralateral to injury) hemisphere. These findings enrich our understanding of the cerebral metabolism mechanisms of electroacupuncture from the perspective of aging and provide new evidence for developing age-specific rehabilitation strategies to improve the efficacy of clinical treatment.

## Data availability statement

The raw data supporting the conclusions of this article will be made available by the authors, without undue reservation.

## Ethics statement

The animal study was reviewed and approved by the Animal Ethical Committee of Shanghai University of Traditional Chinese Medicine.

## Author contributions

J-GX: conceptualization, study design, and supervision. B-BH: data curation, writing—original draft, and writing—review and editing. M-XZ: writing—original draft and writing—review and editing. X-YH: conceptualization and data curation. J-JW and X-XX: methodology and software. JM: methodology and validation. MF: supervision. All authors contributed to the article and approved the submitted version.

## Footnotes

1
http://www.fil.ion.ucl.ac.uk/spm

## References

[B1] Bland D. Zampieri C. Damiano D. (2011). Effectiveness of physical therapy for improving gait and balance in individuals with traumatic brain injury: A systematic review. *Brain Inj.* 25 664–679. 10.3109/02699052.2011.576306 21561297 PMC3319122

[B2] Brehmer Y. Li S. Müller V. von Oertzen T. Lindenberger U. (2007). Memory plasticity across the life span: Uncovering children’s latent potential. *Dev. Psychol.* 43 465–478. 10.1037/0012-1649.43.2.465 17352553

[B3] Calautti C. Leroy F. Guincestre J. Marié R. Baron J. (2001). Sequential activation brain mapping after subcortical stroke: Changes in hemispheric balance and recovery. *Neuroreport* 12 3883–3886. 10.1097/00001756-200112210-00005 11742203

[B4] Chang Q. Lin Y. Hsieh C. (2018). Acupuncture and neuroregeneration in ischemic stroke. *Neural Regen. Res*. 13 573–583. 10.4103/1673-5374.230272 29722298 PMC5950656

[B5] Chavez L. Huang S. MacDonald I. Lin J. Lee Y. Chen Y. (2017). Mechanisms of acupuncture therapy in ischemic stroke rehabilitation: A literature review of basic studies. *Int. J. Mol. Sci.* 18:2270. 10.3390/ijms18112270 29143805 PMC5713240

[B6] Chen A. Lin Z. Lan L. Xie G. Huang J. Lin J. (2012). Electroacupuncture at the Quchi and Zusanli acupoints exerts neuroprotective role in cerebral ischemia-reperfusion injured rats via activation of the PI3K/Akt pathway. *Int. J. Mol. Med.* 30 791–796. 10.3892/ijmm.2012.1074 22842715

[B7] Chollet F. (2013). Pharmacologic approaches to cerebral aging and neuroplasticity: Insights from the stroke model. *Dialogues Clin. Neurosci.* 15 67–76. 10.31887/DCNS.2013.15.1/fchollet 23576890 PMC3622470

[B8] Corrigan J. Cuthbert J. Harrison-Felix C. Whiteneck G. Bell J. Miller A. (2014). US population estimates of health and social outcomes 5 years after rehabilitation for traumatic brain injury. *J. Head Trauma Rehabil.* 29 E1–E9. 10.1097/HTR.0000000000000020 24495919

[B9] Cross D. Garwin G. Cline M. Richards T. Yarnykh V. Mourad P. (2015). Paclitaxel improves outcome from traumatic brain injury. *Brain Res.* 1618 299–308. 10.1016/j.brainres.2015.06.006 26086366 PMC4767255

[B10] Engeroff T. Füzéki E. Vogt L. Fleckenstein J. Schwarz S. Matura S. (2018). Is objectively assessed sedentary behavior, physical activity and cardiorespiratory fitness linked to brain plasticity outcomes in old age? *Neuroscience* 388 384–392. 10.1016/j.neuroscience.2018.07.050 30077618

[B11] En-Tao L. Shu-Xia W. Yong H. Xin-Sheng L. Chun-Zhi T. Shao-Yang C. (2013). Effect of needling at waiguan (SJ5) on brain glucose metabolism in patients with cerebral infarction. *Zhongguo Zhong Xi Yi Jie He Za Zhi* 33 1345–1351. 24432677

[B12] Fang Z. Ning J. Xiong C. Shulin Y. (2012). Effects of electroacupuncture at head points on the function of cerebral motor areas in stroke patients: A PET study. *Evid Based Complement. Alternat Med.* 2012 1–9. 10.1155/2012/902413 22956979 PMC3432396

[B13] Faulkner J. Larkin L. Claflin D. Brooks S. (2007). Age-related changes in the structure and function of skeletal muscles. *Clin. Exp. Pharmacol. Physiol*. 34 1091–1096. 10.1111/j.1440-1681.2007.04752.x 17880359

[B14] Feng Q. Bin L. Zhai Y. Xu M. Liu Z. Peng W. (2019). Long-term efficacy and safety of electroacupuncture on improving MMSE in patients with Alzheimer’s disease. *Zhongguo Zhen Jiu* 39 3–8. 10.13703/j.0255-2930.2019.01.001 30672248

[B15] Feydy A. Carlier R. Roby-Brami A. Bussel B. Cazalis F. Pierot L. (2002). Longitudinal study of motor recovery after stroke: Recruitment and focusing of brain activation. *Stroke* 33 1610–1617. 10.1161/01.str.0000017100.68294.52 12053000

[B16] García-Panach J. Lull N. Lull J. Ferri J. Martínez C. Sopena P. (2011). A voxel-based analysis of FDG-PET in traumatic brain injury: Regional metabolism and relationship between the thalamus and cortical areas. *J. Neurotrauma* 28 1707–1717. 10.1089/neu.2011.1851 21770759

[B17] GBD 2016 Traumatic Brain Injury and Spinal Cord Injury Collaborators (2019). Global, regional, and national burden of traumatic brain injury and spinal cord injury, 1990-2016: A systematic analysis for the Global Burden of Disease Study 2016. *Lancet Neurol.* 18 56–87. 10.1016/S1474-4422(18)30415-0 30497965 PMC6291456

[B18] Goble D. Coxon J. Wenderoth N. Van Impe A. Swinnen S. (2009). Proprioceptive sensibility in the elderly: Degeneration, functional consequences and plastic-adaptive processes. *Neurosci. Biobehav. Rev.* 33 271–278. 10.1016/j.neubiorev.2008.08.012 18793668

[B19] Grady C. (2012). The cognitive neuroscience of ageing. *Nat. Rev. Neurosci.* 13 491–505. 10.1038/nrn3256 22714020 PMC3800175

[B20] Gu T. Wang X. Yang H. She X. Chen K. Wu T. (2020). Impacts of electroacupuncture on neurological function and protein expressions of apoptosis-related Cyt-C and Caspase-9 in rats with traumatic brain injury. *Zhongguo Zhen Jiu* 40 749–755. 10.13703/j.0255-2930.20190521-0001 32648400

[B21] Hua X. Qiu Y. Wang M. Zheng M. Li T. Shen Y. (2016). Enhancement of contralesional motor control promotes locomotor recovery after unilateral brain lesion. *Sci. Rep.* 6:18784. 10.1038/srep18784 26732072 PMC4702126

[B22] Jones S. Davis N. Tyson S. F. (2018). A scoping review of the needs of children and other family members after a child’s traumatic injury. *Clin. Rehabil.* 32 501–511. 10.1177/0269215517736672 29082778 PMC5865473

[B23] Kim Y. Jang S. Byun W. Han B. Lee K. Ahn S. (2004). Ipsilateral motor pathway confirmed by combined brain mapping of a patient with hemiparetic stroke: A case report. *Arch. Phys. Med. Rehabil.* 85 1351–1353. 10.1016/j.apmr.2003.08.102 15295764

[B24] Kim Y. Jang S. Chang Y. Byun W. Son S. Ahn S. (2003). Bilateral primary sensori-motor cortex activation of post-stroke mirror movements: An fMRI study. *Neuroreport* 14 1329–1332. 10.1097/01.wnr.0000078702.79393.9b 12876467

[B25] Koopmans G. Deumens R. Honig W. Hamers F. Steinbusch H. Joosten E. (2005). The assessment of locomotor function in spinal cord injured rats: The importance of objective analysis of coordination. *J. Neurotrauma* 22 214–225. 10.1089/neu.2005.22.214 15716628

[B26] Li F. Sun Q. Shao X. Xie J. Liu H. Xu Y. (2019). Electroacupuncture combined with PNF on proprioception and motor function of lower limbs in stroke patients: A randomized controlled trial. *Zhongguo Zhen Jiu* 39 1034–1040. 10.13703/j.0255-2930.2019.10.002 31621252

[B27] Li Y. Gu J. Zhou J. Xia X. Wang K. Zheng X. (2015). The epidemiology of traumatic brain injury in civilian inpatients of Chinese Military Hospitals, 2001-2007. *Brain Inj.* 29 981–988. 10.3109/02699052.2014.989405 25915805

[B28] Liang S. Jiang X. Zhang Q. Duan S. Zhang T. Huang Q. (2018). Abnormal metabolic connectivity in rats at the acute stage of ischemic stroke. *Neurosci. Bull.* 34 715–724. 10.1007/s12264-018-0266-y 30083891 PMC6129253

[B29] Liu J. Xue X. Wu Y. Yang C. Li N. Li H. (2018). Efficacy and safety of electro-acupuncture treatment in improving the consciousness of patients with traumatic brain injury: Study protocol for a randomized controlled trial. *Trials* 19:296. 10.1186/s13063-018-2687-3 29843761 PMC5975471

[B30] Liu Z. Ng C. Shiu H. Wong H. Chin W. Zhang J. (2018). Neuroprotective effect of Da Chuanxiong formula against cognitive and motor deficits in a rat controlled cortical impact model of traumatic brain injury. *J. Ethnopharmacol.* 217 11–22. 10.1016/j.jep.2018.02.004 29425850

[B31] Mao L. Lv F. Yang W. Zhang T. Li Z. Li D. (2020). Effects of Baihui electroacupuncture in a rat model of depression. *Ann. Transl. Med.* 8:1646. 10.21037/atm-20-7459 33490158 PMC7812171

[B32] Marklund N. Bellander B. Godbolt A. Levin H. McCrory P. Thelin E. (2019). Treatments and rehabilitation in the acute and chronic state of traumatic brain injury. *J. Intern. Med.* 285 608–623. 10.1111/joim.12900 30883980 PMC6527474

[B33] Neumann M. Wang Y. Kim S. Hong S. Jeng L. Bilgen M. (2009). Assessing gait impairment following experimental traumatic brain injury in mice. *J. Neurosci. Methods* 176 34–44. 10.1016/j.jneumeth.2008.08.026 18805438 PMC2588469

[B34] O’Neil-Pirozzi T. Ketchum J. Hammond F. Philippus A. Weber E. Dams-O’Connor K. (2018). Physical, cognitive, and psychosocial characteristics associated with mortality in chronic TBI survivors: A national institute on disability, independent living, and rehabilitation research traumatic brain injury model systems study. *J. Head Trauma Rehabil.* 33 237–245. 10.1097/HTR.0000000000000365 29271788

[B35] Oujamaa L. Relave I. Froger J. Mottet D. Pelissier J. (2009). Rehabilitation of arm function after stroke. Literature review. *Ann. Phys. Rehabil. Med.* 52 269–293. 10.1016/j.rehab.2008.10.003 19398398

[B36] Passow S. Specht K. Adamsen T. Biermann M. Brekke N. Craven A. (2015). Default-mode network functional connectivity is closely related to metabolic activity. *Hum. Brain Mapp.* 36 2027–2038. 10.1002/hbm.22753 25644693 PMC5006878

[B37] Pelled G. Chuang K. Dodd S. Koretsky A. (2007). Functional MRI detection of bilateral cortical reorganization in the rodent brain following peripheral nerve deafferentation. *Neuroimage* 37 262–273. 10.1016/j.neuroimage.2007.03.069 17544301 PMC2253720

[B38] Pitzer C. Kuner R. Tappe-Theodor A. (2016). EXPRESS: Voluntary and evoked behavioral correlates in neuropathic pain states under different housing conditions. *Mol. Pain* 12:1744806916656635. 10.1177/1744806916656635 PMC4956152 27306409

[B39] Rossini P. Calautti C. Pauri F. Baron J. (2003). Post-stroke plastic reorganisation in the adult brain. *Lancet Neurol.* 2 493–502. 10.1016/s1474-4422(03)00485-x 12878437

[B40] Schaechter J. Connell B. Stason W. Kaptchuk T. Krebs D. Macklin E. (2007). Correlated change in upper limb function and motor cortex activation after verum and sham acupuncture in patients with chronic stroke. *J. Altern. Complement. Med.* 13 527–532. 10.1089/acm.2007.6316 17604556

[B41] Shen L. Miao J. Yuan F. Zhao Y. Tang Y. Wang Y. (2013). Overexpression of adiponectin promotes focal angiogenesis in the mouse brain following middle cerebral artery occlusion. *Gene Ther.* 20 93–101. 10.1038/gt.2012.7 22357512

[B42] Sperry M. Kartha S. Granquist E. Winkelstein B. (2018). Inter-subject FDG PET brain networks exhibit multi-scale community structure with different normalization techniques. *Ann. Biomed. Eng.* 46 1001–1012. 10.1007/s10439-018-2022-x 29644496 PMC5980783

[B43] Tambalo S. Peruzzotti-Jametti L. Rigolio R. Fiorini S. Bontempi P. Mallucci G. (2015). Functional magnetic resonance imaging of rats with experimental autoimmune encephalomyelitis reveals brain cortex remodeling. *J. Neurosci.* 35 10088–10100. 10.1523/JNEUROSCI.0540-15.2015 26157006 PMC4495237

[B44] Vella M. Crandall M. Patel M. (2017). Acute management of traumatic brain injury. *Surg. Clin. North Am.* 97 1015–1030. 10.1016/j.suc.2017.06.003 28958355 PMC5747306

[B45] Wang F. Sun L. Zhang X. Jia J. Liu Z. Huang X. (2015). Effect and potential mechanism of electroacupuncture add-on treatment in patients with Parkinson’s disease. *Evid Based Complement. Alternat. Med.* 2015:692795. 10.1155/2015/692795 26351515 PMC4550783

[B46] Wang W. Zhang X. Ji X. Ye Q. Chen W. Ni J. (2015). Mirror neuron therapy for hemispatial neglect patients. *Sci. Rep.* 5:8664. 10.1038/srep08664 25727354 PMC4345335

[B47] Wu X. Hu J. Zhuo L. Fu C. Hui G. Wang Y. (2008). Epidemiology of traumatic brain injury in eastern China, 2004: A prospective large case study. *J. Trauma* 64 1313–1319. 10.1097/TA.0b013e318165c803 18469656

[B48] Xiao L. Wang X. Yang Y. Yang J. Cao Y. Ma S. (2018). Applications of acupuncture therapy in modulating plasticity of central nervous system. *Neuromodulation* 21 762–776. 10.1111/ner.12724 29111577

[B49] Zhang J. Mitsis E. Chu K. Newmark R. Hazlett E. Buchsbaum M. (2010). Statistical parametric mapping and cluster counting analysis of [18F] FDG-PET imaging in traumatic brain injury. *J. Neurotrauma* 27 35–49. 10.1089/neu.2009.1049 19715400

